# The effect of different high-heel types on muscle activation of the paraspinal muscles during standing

**DOI:** 10.1186/1757-1146-7-S1-A116

**Published:** 2014-04-08

**Authors:** Dongwook Han

**Affiliations:** 1 Department of Physical Therapy, Silla University, 700 Beon-gil, Baegyang-daero, Sasang-gu, Busan, 617-736, Republic of Korea

## Purpose

This study researched the effects of high heels which are same in height that is 8cm, but different in types that are Wedge heel, Setback heel and French heel on muscle activation of the paraspinal muscles surrounding cervical, thoracic and lumbar spine.

## Subject

The 28 subjects of this study were females in their 20s, with a foot size of 225~230mm and a normal gait pattern, who had no foot deformities or muscle problems. They voluntarily signed a consent form after hearing the experiment methods.

## Methods

To measure the muscle activations of the C6, T7 and L5 paraspinal muscles and lumbar multifidus during standing, EMG (Keypoint, Medrtonic, USA) was used. After breathing was calmed down, muscle activation during standing on the ground with bare foot was measured. Subsequently, muscle activation during standing wearing shoes with 8cm Wedge heel, Setback heel and French heel were measured. The average values of three measurements were used for analysis.

## Result

In the result of examining the effects of heel types, muscle activation of cervical paraspinal muscle induced by Wedge heel, Setback heel and French heel was significantly higher than being on bare foot. However, there was no difference significantly between the heel types. Muscle activation of lumbar paraspinal muscle induced by Wedge heel, Setback heel and French heel was significantly higher than being on bare foot. However, there was no difference significantly between the heel types.

## Conclusions

The height of the heels is a more important variable than the width of the heels about the change of muscle activation of cervical and lumbar paraspinal muscle. So, wearing high-heeled shoes is not recommended to those who have pain and dysfunction in cervical and lumbar region.

